# Supplementary material for: A Compound Containing Aldehyde Dehydrogenase Relieves the Effects of Alcohol Consumption and Hangover Symptoms in Healthy Men: An Open-Labeled Comparative Study
Source: Pharmaceuticals (Basel). 2024 Aug 20;17(8):1087. doi: 10.3390/ph17081087 (PMC11357502; doi:10.3390/ph17081087)
Supplement: Supplementary file 1 [file pharmaceuticals-17-01087-s001.zip › pharmaceuticals-3139096-supplementary.pdf]

A compound containing aldehyde dehydrogenase relieves the effects of alcohol consumption and hangover symptoms in healthy men: An open-labeled comparative study

In-Kyung Jeong[a†*] Anna Han[b,c†], Ji Eun Jun[a], You-Cheol Hwang[a], Kyu Jeung Ahn[a], Ho Yeon Chung[a], Bo Seung Kang[d], and Se-Young Choung[e,f*].

[a]Division of Endocrinology and Metabolism, Department of Internal Medicine, Kyung Hee University Hospital at Gangdong, Kyung Hee University School of Medicine, Seoul, Korea

[b]Department of Food Science and Human Nutrition, Jeonbuk National University, Jeonju, Korea.

[c]K-Food Research Center, Jeonbuk National University, Jeonju, Korea.

[d]Department of emergency Medicine, Hanyang University Guri Hospital, Hangyang University College of Medicine, Guri, Korea.

[e]Department of Preventive Pharmacy and Toxicology, College of Pharmacy, Kyung Hee University, 26, Kyungheedae-ro, Dongdaemun-gu, Seoul, Korea.

[f]Department of Pharmacy, College of Pharmacy, Dankook University, Cheonan, Korea.

† These authors contributed equally to this work.

***Co-corresponding author**: In-Kyung Jeong, M.D., Ph.D. Division of Endocrinology and Metabolism, Department of Internal Medicine, Kyung Hee University Hospital at Gangdong, #892 Dongnam-ro, Gangdong-gu, Seoul, Korea 05278. E-mail: jik1016@naver.com, jik1016@khu.ac.kr Se-Young Choung, Ph.D. Department of Pharmacy, College of Pharmacy, Dankook University, 119, Dandae-ro, Dongnam-gu, Cheonan-si, Chungnaml, Korea. 31116. E-mail: sychoung@khu.ac.kr

Running title: Examination of anti-hangover effects of fermented rice powder-based substances and its safety in health adult subjects

Key words: alcohol drinking, genetic polymorphism, alcohol dehydrogenase, aldehyde dehydrogenase

Supplementary Table S1. Composition of KISLip®

Ingredients	Content (mg)	Combination ratio (%)
**Low KISLip®** 1 tablet= 2000mg (=2,000mg/tablet x 1, fermented rice powder 500mg)		
Fermented Rice Powder	500	25.00
Refined Glucose	1054.94	53.347
Citric acid	140	1.050
Vitamin C	40	2.000
Erythritol	30.2	1.510
Vitamin B1	0.24	0.012
Vitamin B2	0.28	0.014
Calcium pantothenate	0.12	0.006
Honey powder	84	4.200
Biotin	0.02	0.001
Vitamin A	1.8	0.090
Nicotinic acid amide	3	0.150
Fish collagen	2	0.100
L-Asparagine	30	1.500
Hovenia Ducis Extract powder	24.8	1.240
Blueberry concentrate	140	7.000
Blueberry flavor powder	46	2.300
Sucrose fatty acid	8	0.400
Acesulfame potassium	0.6	0.030
Sucralose	1	0.050
**Total**	**2,000**	**100**

Ingredients	Content (mg)	Combination ratio (%)
**High KISLip®** 4 tablet= 3000mg (=750mg/tablet x 4, fermented rice powder 1,500mg)		
Fermented Rice Powder	375	50.00
Crystalline glucose	150	20.00
Xylitol	105.38	14.05
Citric acid	18.75	2.50
DL-methionine	7.5	1.00
Vitamin C	7.5	1.00
Enzymatically modified stevia glucosyl stevia	6.375	0.85
5 types of mixed Lactobacillus	0.75	0.10
Apple flavored powder	33.75	4.50
Apple flavor powder	30	4.00
Magnesium stearate	7.5	1.00
Silicon dioxide	7.5	1.00
**Total**	**750**	**100**

Supplementary Table S2. Questionnaire of hangover symptoms.

No.	Symptom	Experience (Yes or No)	Severity				
			5	4	3	2	1
1	Excessive thirsty						
2	Sleepiness						
3	Headache						
4	Dizziness						
5	Vomiting						
6	Fatigue						
7	Stomachache						
8	Nausea						
9	Loss of concentrating						
10	Increase of sensitivity (i.e., brightness and noisy)						
11	Insomnia						
12	Sweating						
13	Depression						
14	Memory break						
15	Others ( )						

1, none; 2, mild; 3, slightly severe; 4, severe; and 5, very severe.
